# Sudden Event Monitoring of Civil Infrastructure Using Demand-Based Wireless Smart Sensors

**DOI:** 10.3390/s18124480

**Published:** 2018-12-18

**Authors:** Yuguang Fu, Tu Hoang, Kirill Mechitov, Jong R. Kim, Dichuan Zhang, Billie F. Spencer

**Affiliations:** 1 Department of Civil and Environmental Engineering, University of Illinois at Urbana-Champaign, Urbana, IL 61801, USA; tuhoang2@illinois.edu (T.H.); bfs@illinois.edu (B.F.S.J.); 2 Department of Computer Science, University of Illinois at Urbana-Champaign, Urbana, IL 61801, USA; mechitov@illinois.edu; 3 Department of Civil and Environmental Engineering, Nazarbayev University, Astana 010000, Kazakhstan; jong.kim@nu.edu.kz (J.R.K.); dichuan.zhang@nu.edu.kz (D.Z.)

**Keywords:** sudden event monitoring, wireless smart sensors, demand-based nodes, event-triggered sensing, data fusion

## Abstract

Wireless smart sensors (WSS) have been proposed as an effective means to reduce the high cost of wired structural health monitoring systems. However, many damage scenarios for civil infrastructure involve sudden events, such as strong earthquakes, which can result in damage or even failure in a matter of seconds. Wireless monitoring systems typically employ duty cycling to reduce power consumption; hence, they will miss such events if they are in power-saving sleep mode when the events occur. This paper develops a *demand-based WSS* to meet the requirements of sudden event monitoring with minimal power budget and low response latency, without sacrificing high-fidelity measurements or risking a loss of critical information. In the proposed WSS, a programmable event-based switch is implemented utilizing a low-power trigger accelerometer; the switch is integrated in a high-fidelity sensor platform. Particularly, the approach can rapidly turn on the WSS upon the occurrence of a sudden event and seamlessly transition from low-power acceleration measurement to high-fidelity data acquisition. The capabilities of the proposed WSS are validated through laboratory and field experiments. The results show that the proposed approach is able to capture the occurrence of sudden events and provide high-fidelity data for structural condition assessment in an efficient manner.

## 1. Introduction

Many civil infrastructure damage scenarios involve sudden events, such as natural disasters (e.g., earthquakes) and human-induced hazards (e.g., collisions, explosions, acts of terrorism). The occurrence of these events is generally unpredictable, and the consequences can be catastrophic. A typical example of catastrophic sudden event is found in the accidental collision between barges and a piling of railroad bridge in Mobile, Alabama, in 1993 [1]. As a result, the railroad bridge was damaged and gave way 20 min later when an Amtrak train crossed, killing 47 people. If this collision had been detected immediately and timely structural assessment of the bridge made, then the deaths of these individuals may have been prevented.

To mitigate the consequences of sudden events, the development of monitoring systems is of great importance. Traditional monitoring systems use wired sensors [2,3,4,5]. These monitoring systems not only enable sudden event detection but can also facilitate rapid condition assessment of civil infrastructure. Such wired monitoring systems require line power to operate and can be expensive for many large-scale structures, due primarily to high installation costs [6], often ranging from $5 K to $20 K per channel (e.g., the Bill Emerson Memorial Bridge monitoring system cost a total of $1.3 M for 86 sensors [4,7]).

High-fidelity wireless sensors offer tremendous opportunities to reduce costs and realize the promise of pervasive sensing for structural condition assessment. However, sudden event detection using wireless sensors remains elusive. For example, the monitoring system installed on the Golden Gate Bridge was unable to detect the three earthquakes that occurred during the three-month monitoring deployment [8]. Two main challenges to detect sudden events are apparent:

(i) Limited power. Most wireless sensors are duty-cycled to preserve limited battery power; as a result, wireless sensors will miss the occurrence of sudden events when they are in power-saving sleep mode. Because the duty cycle is typically below 5% [9], this scenario is quite likely to occur.
(ii) Response latency. Response of wireless smart sensors (WSS) from sleep mode to data acquisition may take over a second, resulting in the loss of critical information in short-duration events (e.g., earthquakes and collisions). Moreover, even if awake, sensors may be busy with other tasks (e.g., data transmission); therefore, they will be unable to respond immediately to the occurrence of sudden events, and hence miss the short-duration events.

Addressing these challenges is critical to realizing a WSS for sudden event detection.

One intuitive strategy is to provide sustainable power for WSS to enable continuous monitoring of structures subjected to sudden events, emulating traditional wired monitoring systems. For example, in 2011, Potenza et al. [10] installed a wireless structural health monitoring (SHM) system consisting of 17 WSS on a historical church, which was damaged during the 2009 L’Aquila earthquake. The nodes were powered by the existing electrical lines, which guaranteed the continuity of operation and successfully detected several earthquakes over a 3-year monitoring period. Their strategy of using electrical lines to power WSS does not retain the inherent advantages of cable removal, and thus is not practical for other sudden event monitoring applications using WSS. Energy harvesting and wireless power transfer technologies also do not provide an efficient solution. Although technologies such as solar and wind energy harvesting have been developed and validated to power WSS for periodic monitoring [11,12,13], the challenge is that energy harvesting from the ambient environment is intermittent and time-varying, which is not reliable to support continuous monitoring of structures. Radio frequency (RF) energy transfer and harvesting is another wireless power technique in which WSS convert the received RF signals into electricity. The energy can be transferred reliably over a distance from a dedicated energy source to each node, or dynamically exchanged between different nodes [14]. However, the energy harvesting rate is on the order of micro-watts with low efficiency [15] and is insufficient for high-power high-fidelity monitoring of sudden events.

On the other hand, power consumption can be reduced by employing various energy-saving mechanisms, which help to mitigate, but do not fully address the challenge of limited power for WSS. For example, Jalsan et al. [16] proposed layout optimization strategies for wireless sensor networks to prolong the network lifetime by optimizing communication schemes without compromising information quality. Other examples of energy-saving mechanisms include data reduction, radio optimization, and energy-efficient routing. More detailed discussion can be found in Reference [17]. Most of these strategies are designed to reduce power consumption for wireless transmission, which does not help energy conservation for continuous sensing, because most of the power draw comes from the always-on sensor.

Recent developments in event-triggered sensing present both opportunities and challenges to realize sudden event monitoring using WSS. In event-triggered sensing, WSS only initiates measurement in response to signaling of events, which helps to save both energy and memory resources, and thus prolong the lifetime of WSS. Research has been conducted to implement low-power components (sensors and radios) that enable continuous operation and triggering mechanisms inside each sensor node. Lu et al. [18] designed the TelosW platform, which is an upgrade of the TelosB platform [19], by adding ultra-low power wake-on sensors and wake-on radios. The wake-on sensor is able to wake up the microcontroller (MCU) on occurrence of events with a predetermined threshold. Additionally, the wake-on radio can wake up the MCU when a triggering radio message is received. Similarly, Sutton et al. [20] presented a heterogeneous system architecture which included a low-power event detector circuit and low-power wake-up receivers. Although these two technologies achieve low power consumption, they do not satisfy the high-fidelity requirement of sudden event monitoring for civil infrastructure. For example, the TelosW’s analog to digital converter has only 12-bit resolution. Event-triggered sensing is also developed and implemented to facilitate railway bridge monitoring, because strain cycles and vibrations induced by trains are the most important data for bridge condition assessment (e.g., fatigue), but the arrival time of trains is generally unpredictable. Bischoff et al. [21] deployed a wireless monitoring system which provided strain measurement and fatigue assessment of the Keraesjokk Railway Bridge. Each node was triggered independently by a low-power microelectromechanical systems (MEMS) accelerometer which operated continuously and detected an approaching train. Bias due to the transient start-up nature of the strain gage was removed by a post-processing technique. Liu et al. [22] developed an on-demand sensing system, named ECOVIBE, to monitor train-induced bridge vibrations. In each wireless node, a passive event detection circuit was designed to monitor bridge vibration with no power consumption, and another adaptive logical control circuit powered off the node once the designated tasks were finished. While effective for some applications, all the aforementioned approaches will lose critical data between the occurrence of the event and the time that data begins to be collected. Conversely, response times of wireless sensors from a cold boot to data acquisition are typically well over a second, making this problem particularly acute for short-duration sudden events (e.g., impacts can last only fractions of a second).

Moving the triggering mechanism to outside the sensor nodes provides a solution to address the challenge of data loss. In general, a separate trigger node or system is used to monitor the events continuously and notify of events to sensor nodes which are in power-saving mode most of the time. The trigger node/system is required to send notifications with a certain amount of time before the arrival of events at the structure, compensating the response latency of other sensor nodes. For example, an event-driven wireless strain monitoring system was implemented on a riveted steel railway bridge near Wila, Switzerland [23]. Two trigger nodes, referred to as sentinel nodes, were placed at 50 and 85 m away from the bridge, detecting approaching trains and sending alarm messages using a reliable flooding protocol to wake up sensor nodes on the bridge, before the train arrived. In a 47-day deployment, the system successfully detected 99.7% of train-crossing events. Likewise, in order to detect earthquakes and initiate seismic structural monitoring, Hung et al. [24] developed an intelligent wireless sensor network embedded with an earthquake early warning (EEW) system which was able to detect P-waves before earthquakes arrived. In addition, each sensor node was implemented with a wake-on radio which supported ultralow-power periodic listening of wake-up commands, while the main sensor node was in deep sleep mode. Once the P-wave was detected, the gateway node, integrated with the EEW system, sent wake-up commands to sensor nodes approximately 2 s ahead of earthquakes. Subsequently, sensor nodes started measurement with a latency time of only 229 ms. Despite successful detection of train-crossing and seismic events, the aforementioned methods do not provide a universal solution to address the challenge of data loss for many other sudden events, e.g., bridge impact by over-height trucks and ships which can hardly be detected ahead of impacts.

In addition, some progress has been made in addressing the challenge of response latency to sudden events, when WSS are awake but not in sensing mode. Cheng & Pakzad [8] proposed a pulse-based media access control protocol. When an earthquake occurs, a trigger message with high priority is propagated from an observation site across the WSS network to preempt current tasks; sensors will be forced to conduct measurement to capture the structural response under the earthquake. Dorvash et al. [25] developed the Sandwich node to reduce the response latency for unexpected events. A smart trigger node continuously measures the structural response; it will broadcast a proper message across a network of Sandwich nodes in the case of occurrence of events. Sandwich nodes keep listening to the trigger message; they will preempt current tasks once the trigger message is received. Response delay of Sandwich nodes is around 8 ms to the occurrence of events. Although response delay is reduced in these two strategies, the wireless sensor’s radio must always be on to listen for messages from a trigger node. Unless employing an ultralow-power wake-up radio, these strategies will result in a significant power draw.

This paper proposes a new approach for monitoring civil infrastructure subjected to sudden events, aimed at detecting sudden events of any duration and capturing complete transient response of any length. A demand-based wireless smart sensor (WSS) is developed that can capture data during the sudden event that is suitable for rapid condition assessment of civil infrastructure. As opposed to periodic monitoring, the *demand-based WSS* only wakes up and initiates sensing in response to specific conditions, such as sudden events. The results of laboratory experiments and a field experiment show that our proposed approach can capture the occurrence of sudden events and provide high-fidelity data for structural condition assessment in a timely and power-efficient manner.

## 2. Demand-Based WSS

As discussed in the previous section, the primary issues that must be overcome to use wireless sensors to monitor civil infrastructure subjected to sudden events are: (i) the sensor must operate on battery power, (ii) high-fidelity data appropriate for SHM application must be obtained, (iii) data surrounding the occurrence of sudden events must not be lost, and (iv) the WSS node must have sufficient computational power to translate the data collected into actionable information. This section describes a *demand-based WSS* system that can address these issues.

### 2.1. Ultralow-Power Trigger Accelerometer for Continuous Monitoring

To ensure that the occurrence of sudden events is not missed, the monitoring system must be continuously in an on state. A wireless node that is always on would quickly deplete its battery. Therefore, the solution proposed herein is to use an ultralow-power trigger accelerometer that can continuously monitor the vibration of structures; the data from the accelerometer is stored in a First-In-First-Out (FIFO) buffer. When an event occurs, the data in the FIFO buffer will be frozen, and the sensor triggers an interrupt signal to wake up the main sensor platform and start sensing. Such a trigger accelerometer should have low power consumption to enable continuous monitoring for several years, good sensing characteristics, including a high sampling rate and adequate resolution, and a large FIFO buffer to ensure data is not lost after the triggering event.

Trigger accelerometers in the market today were compared and the candidates that satisfied the basic needs of sudden event monitoring are listed in Table 1. The power consumption reported in the table correspond to the ultralow-noise mode of each sensor. More specifically, the ADXL362, developed by Analog Devices, consumes much less power than the other trigger accelerometers. The ADXL372, an updated high-g version of ADXL362, has a larger sampling rate and measurement range, but with a sensing resolution of only 100 mg. The LIS3DSH from STMicromechanics has high resolution of 0.06 mg, but it has a high-power draw and an inadequate FIFO buffer. Finally, the MPU6050 developed by InvenSense features a large FIFO buffer and high resolution, but it consumes substantial power.

**Figure 1:**
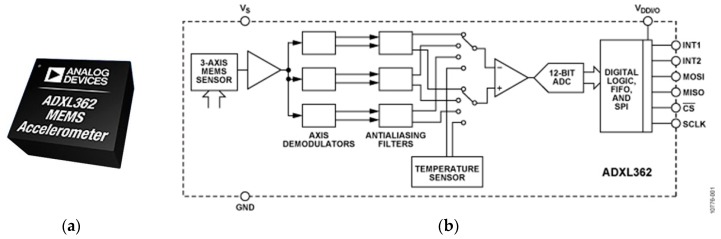
ADXL362: (**a**) sensor chip; (**b**) Functional block diagram [26].

In sum, based on application needs, the ADXL362 has been selected for this study (Figure 1); it integrates a three-axis microelectromechanical systems (MEMS) accelerometer with a temperature sensor, an analog-to-digital converter, and a Serial Peripheral Interface (SPI) digital interface. The ADXL362 consumes only 13 uA in ultralow-noise mode at 3.3 V, which theoretically could work continuously for over two years on a single coin-cell battery. A sampling rate up to 400 Hz and a resolution of 1 mg is supported, satisfying many SHM applications. The large FIFO buffer allows the sensor to save up to 512 samples, which corresponds to 1.7 s for all three axes sampled at 100 Hz. Moreover, it has built-in logic for acceleration threshold detection; a detected event can be used as a trigger to wake up the primary sensor node.

### 2.2. High-Fidelity Sensor Platform for Sudden Event Monitoring

To provide high-quality measurement data and enable rapid condition assessment of structures subjected to sudden events, the sensor platform should have following features: (i) sensors and a data acquisition system that can obtain high-quality data at a high sampling rate for the event; (ii) powerful microcontroller to acquire and analyze sensor data in near real time. Other important features include: reliable communication, open-source software, and efficient data and power management.

A summary of the most advanced wireless sensor platforms available in the market currently is given in Table 2. The Xnode, developed by Embedor Technology, has a 24-bit Analog-to-Digital Converter (ADC), which is the best in its class. The microprocessor unit (MCU) of Waspmote (Libelium, Zaragoza, Spain) is not able to support rapid processing of large amount of data. The MCU information for the AX-3D (BeanAir, Berlin, Germany) and the G-Link-200 (LORD Sensing, Williston, VT, USA) is unavailable, and the operating systems of these platforms are proprietary. Note that several high-performance wireless sensor platforms (e.g., Imote2) are no longer commercially available, and hence not compared herein.

**Figure 2:**
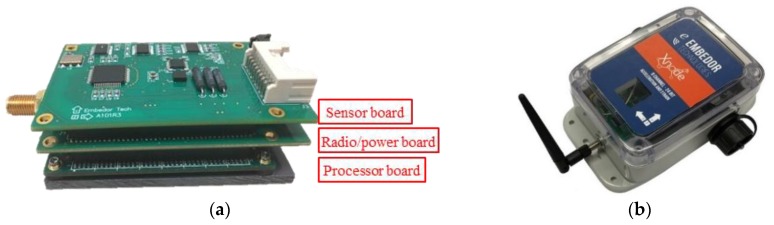
Xnode: (**a**) stacked modular boards; (**b**) weather-proof enclosure [34].

Because of its high sensing resolution, high sampling rate, powerful microprocessor, and open-source software, the Xnode Smart Sensor [34] has been selected as the host wireless sensor platform in this study. The standard Xnode consists of three modular printed circuit boards (PCB): (i) the processor board, (ii) the radio/power board, and (iii) the sensor board (Figure 2). In particular, it employs an 8-channel, 24-bit ADC (Texas Instruments ADS131E8), allowing a maximum sampling rate up to 16 kHz and an NXP LPC4357 microcontroller operating at frequencies up to 204 MHz, which can be used to execute data-intensive on-board computation. Moreover, it implements open-source middleware services [35], which facilitates custom application development. In addition, it possesses two SPI controllers, making it possible to communicate with the selected trigger accelerometer, the ADXL362.

### 2.3. Integration of Wake-up Sensor and High-Fidelity Sensor Platform

To capture the entire event without loss of critical information, the ADXL362 accelerometer and the Xnode must be carefully integrated to build a *demand-based WSS*. This integration is discussed in the remainder of this section, in terms of hardware, software, and digital signal processing.

#### 2.3.1. Hardware Consideration

To address the challenge of physical integration of the ADXL362 accelerometer into the Xnode, a programmable event-based switch was designed and implemented on the radio/power board of the Xnode in the *demand-based WSS*.

When a sudden event occurs, and the vibration exceeds a user-defined threshold, an interrupt pin in the ADXL362 generates a triggering signal. This signal is connected to a MOSFET to flip its state, turning on the Xnode and initiating high-fidelity sensing. When the event ends (lack of acceleration above a threshold), the other interrupt pin in the ADXL362 generates a signal to notify the Xnode to stop high-fidelity sensing. After data acquisition is completed, the triggering signal is cleared, and the MOSFET turns off the Xnode. The communication of control messages between the ADXL362 and the Xnode is carried out via SPI bus through a four-wire connection. In addition to enable event-triggered sensing, the proposed switch should be designed to retain traditional functionality for periodic monitoring. Specifically, sensor nodes are operated on low duty cycles, and the base station can access the network of nodes at random to initiate operations or measurements in the network. To achieve this goal, a real time clock, DS3231, is employed in the proposed switch. When a user-defined period passes or at a specific time of day, the DS3231 sends a triggering signal which flips the MOSFET switch and turn on the Xnode. Then the node remains awake for a short period of time to listen for messages from the base station. Once a command is obtained, the node carries out the required task (e.g., sensing, battery check). The communication of control messages between the DS3231 and the Xnode is conducted through the I2C bus.

**Figure 3:**
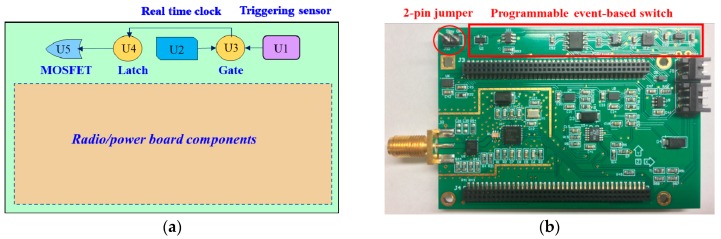
PCB design for the event-based switch: (**a**) design concept (**b**) realized PCB.

Figure 3a illustrates the design concept of the proposed switch. Five major components are implemented, including a trigger sensor ADXL362 (U1), a real time clock DS3231 (U2), an AND gate (U3), a latch (U4), and a MOSFET (U5). Interrupt pins from the ADXL362 and the DS3231 are connected to the MOSFET through the AND gate. This circuit enables the MOSFET to be triggered by either the real time clock or the trigger sensor. In addition, a latch component is added between the AND gate and the MOSFET, to keep the power supply stable. Figure 3b shows the realized PCB for the proposed switch. The five major components, as well as companion resistors and capacitors, are all soldered on the edge of top side. In some use cases such as downloading code to the board and debugging, the sensor platform should always be on and therefore the designed switch needs to be bypassed. To achieve this goal, a 2-pin jumper is added. When the two pins on the jumper are not connected, the proposed switch works as designed, otherwise, it is bypassed.

#### 2.3.2. Software Consideration

In addition to hardware development of the prototype, an effective application framework is required to control the behavior of *demand-based WSS* to realize event-triggered sensing.

**Figure 4:**
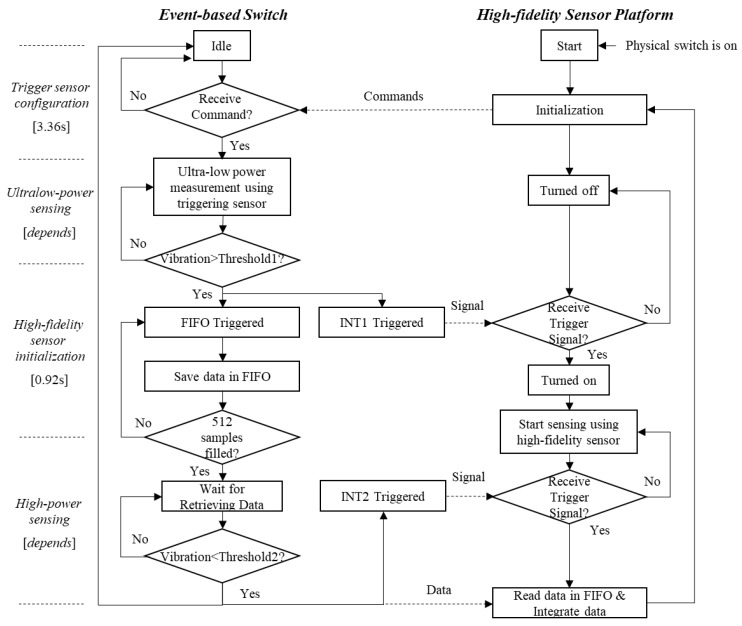
Flowchart of demand-based wireless smart sensors (WSS) for event-triggered sensing.

Figure 4 shows a flowchart of the application framework for the *demand-based WSS*. More specifically, when users turn on the physical switch of a main sensor platform, the Xnode first initializes itself and sends commands which contain configuration parameters (e.g., threshold, timers, and data buffer size) to the event-based switch discussed in the previous section. Once the commands have been received, the switch completes configuration of the device settings. Subsequently, the ADXL362 starts measurement in ultralow-noise mode, and the Xnode is turned off. If a sudden event occurs and the acceleration obtained in the ADXL362 exceeds the user-defined threshold, an interrupt pin, INT1 on the ADXL362 sends a trigger signal to turn on the Xnode. Concurrently, the ADXL362 saves 512 data samples into its FIFO buffer surrounding the onset of the event and waits for the Xnode to retrieve the data. The Xnode starts high-fidelity data acquisition using its built-in high-power high-accuracy MEMS accelerometer. When the event stops and the acceleration obtained in the ADXL362 is lower than a user-defined threshold for a certain period of time, the other interrupt pin, INT2, in the ADXL362 is triggered. Subsequently, the Xnode stops high-fidelity sensing. After sensing is completed, the Xnode reads data from the FIFO buffer of the ADXL362 and fuses it with the Xnode data. In addition, when the Xnode is busy with other tasks (e.g., data transmission of a previous event), but another sudden event occurs, the INT2 pin can be configured to interrupt undergoing tasks and force the Xnode to start high-fidelity sensing immediately. In addition, timing analysis results for each stage of a demand-based WSS are presented in the left of Figure 4.

For sudden events that are rare, e.g., earthquakes, the thresholds can be determined based on priori information about the sudden events that are monitored. The a priori information can be estimated by numerical analysis, the data in the history, or the measurement data in a preliminary test. For some events that occur frequently, e.g., railway bridge impacts from over-height vehicles, the thresholds can be determined adaptively, starting from a relatively low value during a “training phase” and then adjusted until the detection errors are minimized. A more comprehensive research regarding the triggering mechanism is the subject of future work to be addressed in the near future.

#### 2.3.3. Data Fusion of Trigger Sensor and Xnode Acceleration Record

The objective of the *demand-based WSS* is to obtain the data from before the trigger event occurs until the structural accelerations stop. Specifically, the ADXL362 can record structural measurements surrounding the onset of a sudden event, whilst the Xnode starts sampling the data approximately 0.9 s after being triggered. Therefore, the ADXL362 data and the Xnode data must be synchronized and fused to produce a complete representation of the acceleration record. The following paragraphs describe the challenges encountered in this process, along with the associated resolutions.

To fuse the two overlapping data streams, two main challenges should be addressed, including (i) differences in the sampling rate between the ADXL362 and the Xnode, and (ii) synchronization error between the ADXL362 data and the Xnode data. More precisely, the first challenge results from the differences between the clock rates of the ADXL362 and the Xnode. The internal clock rate in the ADXL362 has a standard deviation of approximately 3%. One approach might be to calibrate the ADXL362 incorporated in each Xnode; however, this approach is not practical, as the clock rate will change with temperature, invalidating the initial calibration. The second challenge is due to the variance in start-up time of the sensing task on the Xnode. As a result, a random offset will exist between the two data streams.

**Figure 5:**
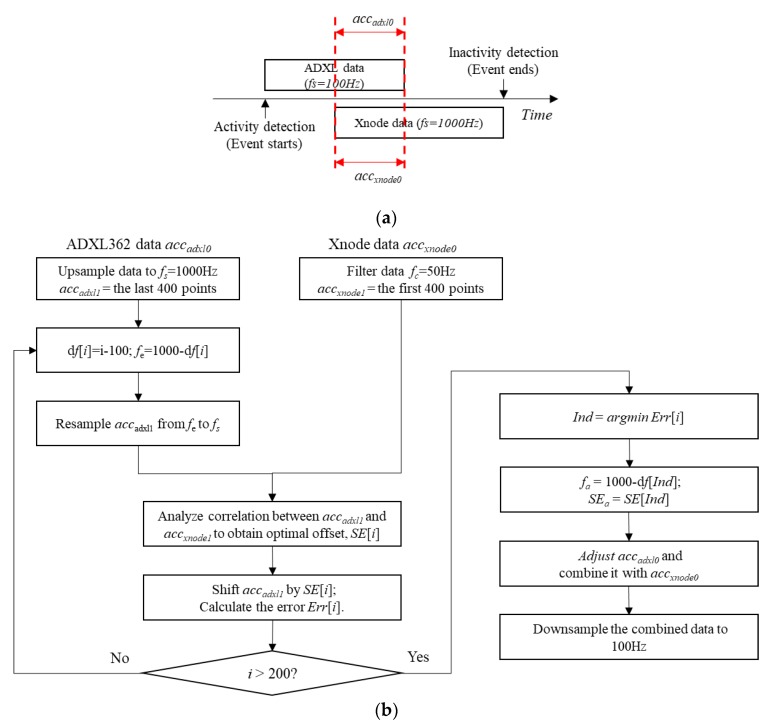
Post-sensing data fusion: (**a**) illustration of two data sources, (**b**) flowchart of data fusion strategy.

To tackle the challenges identified in the previous paragraph, the beginning of the Xnode data, which is overlapped with the ADXL362 data, was utilized to calibrate the entire ADXL362 data stream. Figure 5 shows a flowchart of this approach. More specifically, the ADXL362, $acc_{adxl0}$, with a nominal sampling rate of 100 Hz, were first up-sampled to $f_{s}$ (1000 Hz). The last 400 data points of the $acc_{adxl0}$ were chosen as $acc_{adxl1}$, which was assumed to be approximately overlapped with the beginning of the Xnode data. In the meantime, the Xnode data, $acc_{xnode0}$, was sent through an 8-pole elliptic low-pass filter with a cutoff frequency of 50 Hz, to have the same bandwidth with $acc_{adxl0}$. The first 400 data points of $acc_{xnode0}$ were considered as $acc_{xnode1}$. Based on the datasheet of ADXL362 [26], the clock frequency deviation from the ideal value was within the range of −10% and 10%. Therefore, to find the actual sampling frequency of $acc_{adxl1}$, exhaustive search was applied from 900 Hz to 1100 Hz. For Step *i*, the estimated sampling frequency ($f_{e}$) of the ADXL362 data was set as,

(1) $$f_{e}=1000-df[i]$$

where, df[i]=i−100, i∈[0,200]. $acc_{adxl1}$ was resampled from $f_{e}$ to $f_{s}$ using resampling-based approach [36]. Then, to estimate the synchronization error between $acc_{adxl1}$ and $acc_{xnode1}$, the cross-correlation between the two data segments was calculated. The optimal offset, SE[i], was obtained, for which the cross-correlation reaches its maximum value. Afterwards, $acc_{adxl1}$ was shifted by SE[i], and then the data fusion error (Err) was calculated as,

(2) $$Err[i]={\left\|acc_{adxl1}-acc_{xnode1}\right\|}_{2}$$

where, ${\left\|\;\right\|}_{2}$ represents Euclidean norm. After completing these steps, the best estimations of sampling frequency $f_{a}$ and synchronization error $SE_{a}$ were obtained in the step that achieves minimal Err. Subsequently, $f_{a}$ and $SE_{a}$ were applied to calibrate the original data set, $acc_{adxl0}$. Finally, $acc_{adxl0}$ and $acc_{xnode0}$ were combined and down-sampled to 100 Hz for ensuing analysis.

## 3. Validation of the Demand-Based WSS Performance

To validate the performance of the *demand-based WSS*, laboratory tests were carried out for data fusion and earthquake monitoring. The detailed test setup and results are presented in this section. The performance of the *demand-based WSS* is discussed, in terms of power consumption, sensing characteristics, and data quality for sudden event monitoring.

### 3.1. Validation of Data Fusion

**Figure 6:**
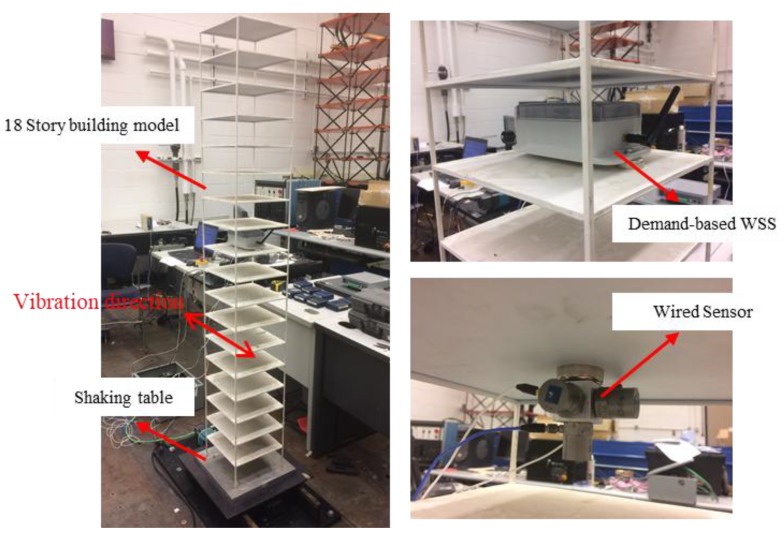
Experiment setup for a *demand-based WSS*.

A lab test was conducted to illustrate the challenge of data fusion between the ADXL362 data and the Xnode data. Specifically, a *demand-based WSS* was located at 10th floor of an 18-story building model shown in Figure 6. The ADXL362 was configured to capture samples at 100 Hz, starting at 0.2 s before the triggered event and continuing until 1.5 s after the event. The event-triggering threshold was set to 150 mg, at which time, the Xnode was turned on and 1000 Hz high-fidelity measurement was started. To reduce false positives, two consecutive data points exceeding the threshold were required to cause triggering. In addition, a wired piezoelectric accelerometer, model PCB353B33, was installed on the same floor and sampled at a frequency of 128 Hz. The acceleration from these sensors served as reference data. A sudden event was simulated by manually shaking the building model in horizontal direction.

**Figure 7:**
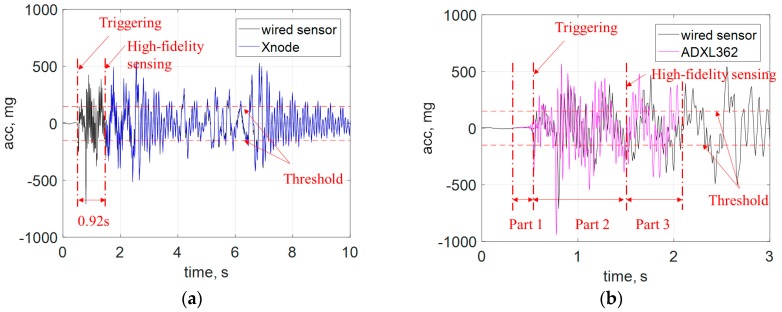
Time history data comparison: (**a**) Xnode measurement, (**b**) ADXL362 data buffer.

To make a direct comparison in the time domain, the Xnode data and wired sensor data were sent through an 8-pole elliptic low-pass filter with a cutoff frequency of 50 Hz, as displayed in Figure 7. The direction of acceleration measurement was the same with the vibration direction specified in Figure 6. The vibration exceeded the threshold at 0.7 s, and the event-based switch turned on the Xnode. The Xnode required 0.92 s for initialization. Fortunately, as shown in Figure 7b, acceleration data stored in the FIFO buffer of the ADXL362 was recorded during this period. Specifically, the ADXL362 data can be divided into three parts: (i) Part 1 is the pre-triggered data which is around 0.28 s in length; (ii) Part 2 is the data that cover the time where the Xnode is initializing; (iii) Part 3 is where the ADXL362 data overlaps with the Xnode data. The length of the data in Part 3 is approximately 0.6 s. As shown in Figure 7b, the data obtained from the ADXL362 does not match well with the reference data from the wired sensors, because the sampling frequency of the ADXL362 was slightly smaller than 100 Hz, which illustrates the first challenge mentioned in the Section 2.3.3. In addition, the time offset between the Xnode data (blue line in Figure 7a) and the ADXL362 data (red line in Figure 7b) must be accurately estimated to fuse these two data streams, which illustrates the second challenge of data fusion.

**Figure 8:**
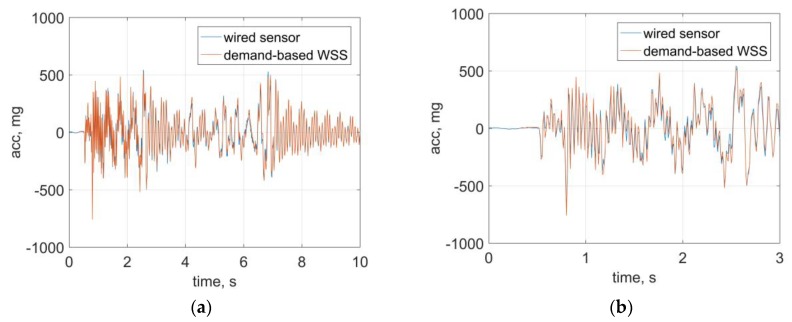
Results of data fusion: (**a**) time history data, (**b**) zoomed view (ADXL362 data).

The data fusion strategy discussed in the previous section is applied to the test data. Figure 8 shows a comparison of time history data between sensor data from wired sensors and the fused data from the *demand-based WSS*. The excellent agreement demonstrates the ability of the proposed strategy to seamlessly capture the structural response subjected to a sudden event.

### 3.2. Earthquake Monitoring

As a typical sudden event, an earthquake is transient and unpredictable, and the consequences can be catastrophic. Continuous efforts are required to develop cost-effective earthquake monitoring systems to mitigate the effect of earthquakes. *Demand-based WSS* have a significant potential to enable earthquake detection and rapid condition assessment of civil infrastructure, which was validated through a lab test in this section.

The test setup was the same with that in Section 3.1, as shown in Figure 6. The structure model was mounted on a uniaxial shaking table. This shaking table can simulate earthquakes in one horizontal direction, driving a 15 kg mass at 2.5 g with a maximum stroke of ±7.5 cm. The El Centro earthquake excitation was generated by the shaking table to represent a sudden event. The detection threshold for the event-based switch in *demand-based WSS* was configured as follows: the onset of event was detected when the acceleration was above 80 mg over 0.02 s, and the end of event was detected when the acceleration was below 40 mg over 5 s. Other configuration parameters are the same with the test in Section 3.1, such as sampling frequencies and filter parameters.

**Figure 9:**
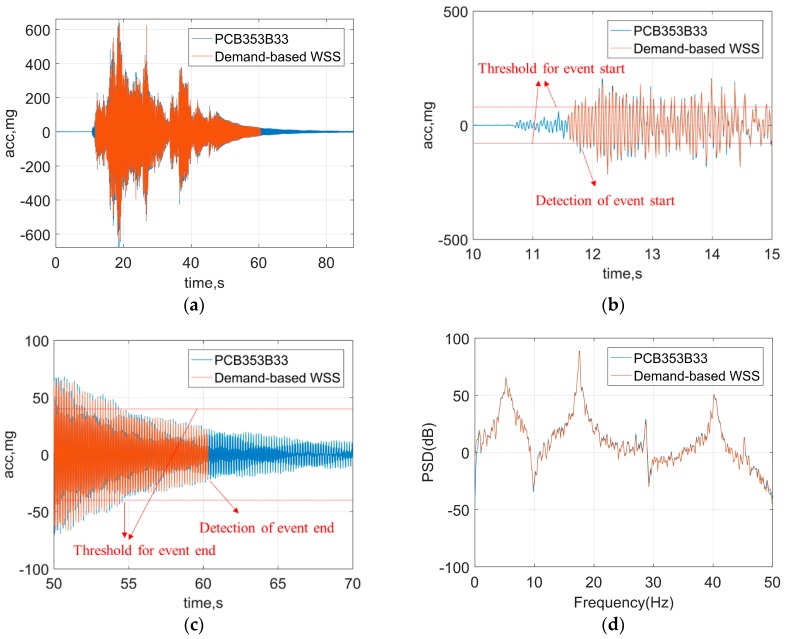
Test results of earthquake monitoring: (**a**) time history data, (**b**) zoomed view of time history data for event start, (**c**) zoomed view of time history data for event end, (**d**) power spectral density (PSD) data.

A segment of 90 s recorded time history is shown in Figure 9a. As can be seen in the zoomed view of the time history data (Figure 9b,c), the ground motion started at 10.7 s, but the vibration in the beginning was very small. From 11.79 s to 11.80 s, two consecutive acceleration samples obtained by the trigger accelerometer exceeded 80 mg. As a result, the event-based switch turned on the *demand-based WSS* immediately and the WSS started high-fidelity measurement. The acceleration became smaller than 40 mg after 54.50 s. Approximately 5 s later, the event-based switch stopped the high-fidelity sensing. Furthermore, Figure 9d shows the power spectral density (PSD) in the frequency domain. The excellent agreement between the results of wired sensors and the *demand-based WSS* in the both time and frequency domain demonstrates the ability of the proposed WSS to detect the earthquake and capture the accurate structural response during earthquakes in a timely and efficient manner.

### 3.3. Evaluation and Discussion

In the lab tests described in previous sections, the attractive performance of the *demand-based WSS* was successfully validated to detect sudden events and provide high-quality sensing data for SHM analysis.

**Figure 10:**
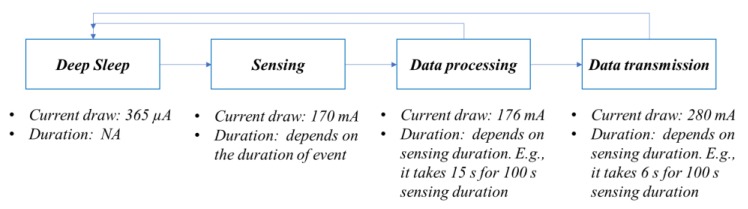
Flowchart of event-triggered sensing regarding current draw and duration for each operation.

(1) Power consumption tests showed that the proposed WSS has a current draw of only 365 µA when no sudden event occurred, but the power consumption of the original Xnode sensor platform is approximately 170 mA during sensing. Considering that sudden events are rare and short-duration, most of the time the demand-based WSS deployed on a structure is in low-power measurement mode. Therefore, if using a 3.7 V DC, 10,000 mAh, rechargeable lithium polymer battery, employing the proposed WSS can extend the lifetime of always-on monitoring from three days to over three years using a single lithium battery. This feature helps to successfully detect the occurrence of sudden events with minimal power budget in long-term monitoring. In addition, the current draw in each operation associated with duration for a demand-based WSS was determined experimentally and shown in Figure 10, in which the majority component of power consumption is sensing.
(2) The data obtained from the *demand-based WSS* is high-quality, matching well with the data from wired piezoelectric accelerometers. In particular, high-fidelity sensing enables 24-bit sensing resolution and over 1 kHz sampling rates. This feature helps to conduct structural condition assessments accurately under sudden events.
(3) The test results show that, when an event occurs, a seamless transition from the low-power sensing to high-fidelity measurement is carried out, without losing any data about the event.

These three features demonstrate that the proposed WSS satisfies the demands of sudden event monitoring.

## 4. Field Application

**Figure 11:**
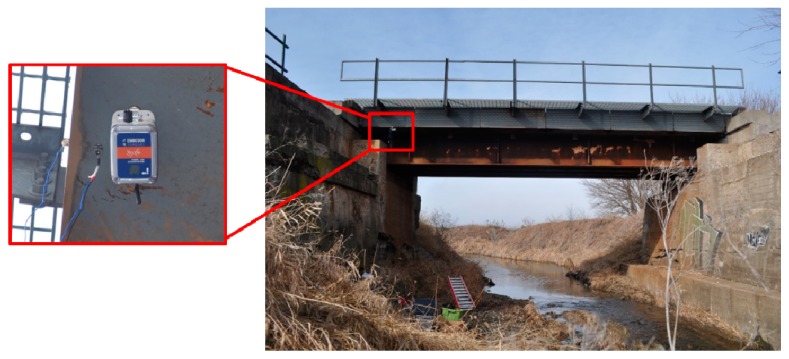
Field application of the *demand-based WSS*.

To further validate system performance, a field test was conducted on a steel railroad bridge north of Champaign, Illinois. Having vibration data while in-service trains traverse the bridge is useful to assess the bridge condition [37]. Train events have similar features to sudden natural events, e.g., unpredictability due to uncertain train schedules, but occur more frequently and therefore provide a convenient test platform. A *demand-based WSS* was deployed on the bottom side of a bridge girder. Simultaneously, wired sensors, model PCB353B33, were selected as reference sensors and deployed close to the WSS (see Figure 11). A detection threshold was configured to be the same as the test in Section 3.2. To avoid signal saturation, the measurement range of the trigger accelerometer was set to the maximum value of 8 g. At 10:52:06 a.m. on 7 May 2019, an Amtrak passenger train passed by the bridge.

**Figure 12:**
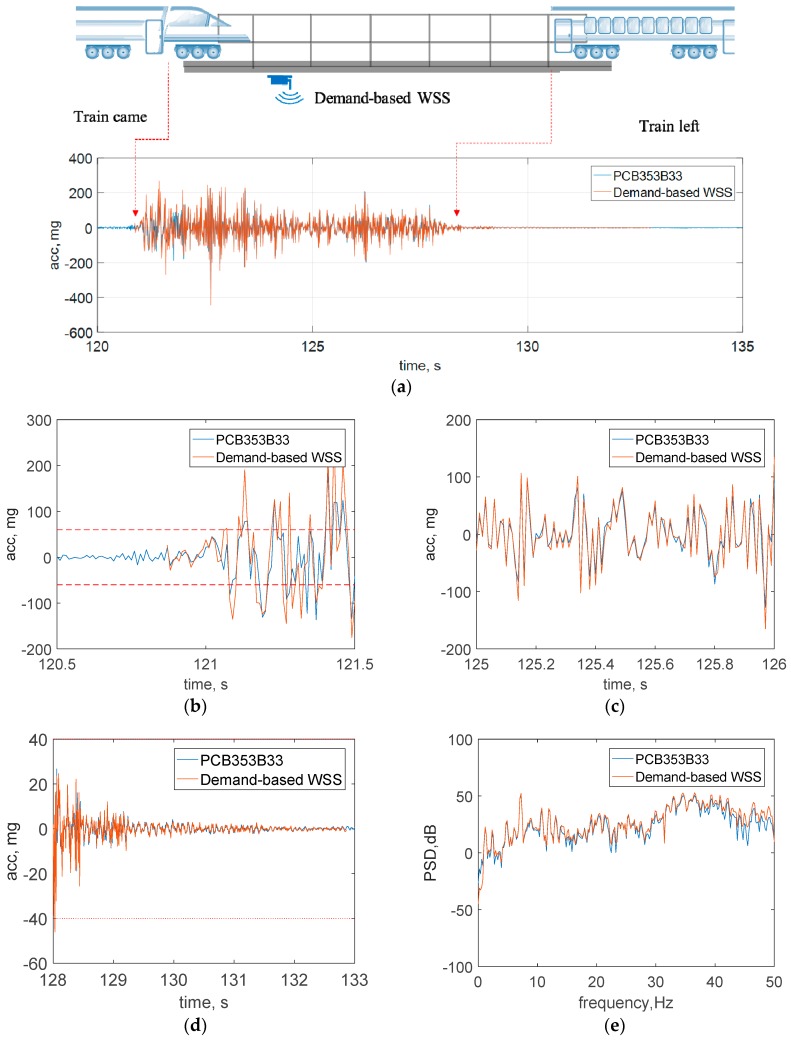
Test results on a railroad bridge: (**a**) time history data, (**b**) zoomed view of event starts (ADXL362 data), (**c**) zoomed view of event data (Xnode data), (**d**) zoomed view of event ends (Xnode data), (**e**) PSD data.

Figure 12a–d shows the raw acceleration data of the bridge in vertical direction. The train came to the bridge at 121 s and left at 128 s. The event had a short duration of 7 s, and it was successfully detected by the *demand-based WSS*. Figure 12e shows the PSD data. Some slight discrepancies between the data from two sensors are possibly due to the different locations of the sensors. In sum, good agreement can be observed between the wired sensor and the *demand-based WSS* both in the time and frequency domain, demonstrating that the new WSS can capture the sudden event and obtain high-fidelity measurement in real applications.

## 5. Conclusions

This paper presented the design of *demand-based WSS* to meet the application requirements of sudden event monitoring. The proposed WSS mainly consist of a unique programmable event-based switch and a powerful high-fidelity WSS platform. In particular, the event-based switch is built on a trigger accelerometer which allows the new WSS to measure the structural response in ultralow-power in long term, so as not to miss sudden events. In addition, the software of event-triggered sensing and data fusion is implemented. The performance of the proposed WSS is evaluated through the lab tests of earthquake monitoring and a field application on a railroad bridge. The test results show that the proposed WSS can continuously monitor structural response with minimal power budget, and hence detect the occurrence of the sudden event with the smallest delay. Besides detecting sudden events, the proposed WSS have the excellent features of high sampling rates and sensing resolution, which finally helps to provide high-quality data in sudden events for rapid condition assessment of civil infrastructure. Moreover, the proposed WSS are powerful and versatile not only for sudden events (e.g., earthquakes), but also for autonomous monitoring of other general events (e.g., bridge/highway overloads).

For large-scale structures, one demand-based WSS is not sufficient, and a network of nodes are needed for a meaningful characterization of the structural response. When subjected to a sudden event, each node may be triggered independently to initiate measurement at slightly different times due to varying response levels in the structure. Future work will address the challenges encountered for a network of demand-based WSS under sudden events. For example, one critical issue is to synchronize data from different sensor nodes without introducing delay of event-triggered sensing.

## Figures and Tables

**Table 1:** Comparison of trigger accelerometers in the market [26,27,28,29].

	ADXL362	ADXL372	LIS3DSH	MPU6050
Manufactures	Analog devices	Analog devices	STMicroelectronics	InvenSense
Supply voltage (V)	1.6–3.5	1.6–3.5	1.7–3.6	2.4–3.5
Power consumption (uA)	13	33	225	500
Sampling rate (Hz)	12.5~400	400~6400	3.125~1600	4~1000
Measurement range (g)	±2, ±4, ±8	±200	±2, ±4, ±8, ±16	±2, ±4, ±8, ±16
Resolution (mg)	1 mg	100 mg	0.06 mg	0.06 mg
Spectral noise (µg/√Hz)	175–350	5300	150	400
Buffer size (samples)	512	512	32	512

**Table 2:** Comparison of most advanced wireless sensor platforms in the market [30,31,32,33,34].

	Martlet	AX-3D	Waspmote	G-Link-200	Xnode
ADC resolution (bits)	12	16	16	20	24
Sampling rate (Hz)	up to 3 M	3.5 k	0.5–1 k	0–4 k	0–1.6 k
MCU Frequency (max. Hz)	80 M	NA	32 k	NA	204 M
LOS range (m)	500	650	1.6 k	2 k	1 k
Operating system	State-machine	Proprietary	Proprietary	Proprietary	FreeRTOS
Data memory	32 GB	1 million data points	16 GB	8 million data points	4 GB
Internal power source	dry cell battery	lithium-ion battery	lithium-ion battery	dry cell battery	lthium-ion battery
External power source	NA	primary cell/8–28 V DC	7 V DC	NA	5 V DC
